# Characterizing Ethiopian cattle production systems for disease burden analysis

**DOI:** 10.3389/fvets.2023.1233474

**Published:** 2023-09-28

**Authors:** Yin Li, Dianne Mayberry, Wudu Jemberu, Peggy Schrobback, Mario Herrero, Gemma Chaters, Theodore Knight-Jones, Jonathan Rushton

**Affiliations:** ^1^Global Burden of Animal Diseases Programme, Liverpool, United Kingdom; ^2^Commonwealth Scientific and Industrial Research Organization, Agriculture and Food, Brisbane, QLD, Australia; ^3^School of Veterinary Medicine and Centre for Biosecurity and One Health, Harry Butler Institute, Murdoch University, Perth, WA, Australia; ^4^International Livestock Research Institute, Addis Ababa, Ethiopia; ^5^Faculty of Veterinary Medicine, University of Gondar, Gondar, Ethiopia; ^6^College of Agriculture and Life Sciences, Cornell University, Ithaca, NY, United States; ^7^Institute of Infection and Global Health, University of Liverpool, Liverpool, United Kingdom

**Keywords:** bovine, herd model, biomass, economic value, Ethiopia, GBADs

## Abstract

This paper addresses knowledge gaps in the biomass, productivity and value of livestock for the pastoral, mixed crop-livestock and specialized dairy systems in Ethiopia. Population size, reproductive performance, mortality, offtake and productivity of cattle were calculated from official statistics and a meta-analysis of data available in the published literature. This information was then used to estimate biomass and output value for 2020 using a herd dynamics model. The mixed-crop livestock system dominates the Ethiopian cattle sector, with 55 million cattle (78% total population) and contributing 8.52 billion USD to the economy through the provision of meat, milk, hides and draft power in 2021. By comparison, the pastoral (13.4 million head) and specialized dairy (1.8 million head) systems are much smaller. Productivity varied between different production systems, with differences in live body weight, productivity and prices from different sources. The estimated total cattle biomass was 14.8 billion kg in 2021, i.e., 11.3 billion kg in the mixed crop-livestock system, 2.60 billion kg in the pastoral system and 0.87 billion kg in the specialized dairy system. The total economic asset values of cattle in the mixed crop-livestock, pastoral and specialized dairy systems were estimated as 24.8, 5.28 and 1.37 billion USD, respectively. The total combined output value (e.g., beef, milk and draft power) of cattle production was 11.9 billion USD, which was 11.2% of the GDP in Ethiopia in 2021. This work quantifies the importance of cattle in the Ethiopian economy. These estimates of herd structure, reproductive performance, productivity, biomass, and economic value for cattle production systems in Ethiopia can be used to inform high-level policy, revealing under-performance and areas to prioritize and provide a basis for further technical analysis, such as disease burden.

## 1. Introduction

Livestock productivity, profitability and sustainability are limited by issues such as animal nutrition, genetics, disease, and management (1). However, a systematic way to estimate losses (such as mortality and loss in production) associated with these issues is lacking. Methods are needed to allow quantification of economic losses associated with livestock disease and other issues that depress livestock productivity, and to attribute these losses to specific causes or syndromes (2). Such information can then be used by policymakers to inform targeted investments in animal health.

Characterizing livestock production systems is key to understanding the burden of animal diseases. The burden of disease varies between livestock production systems due to differences in production purposes, breeds, management, and access to veterinary services. These factors all contribute to the risk of disease and the magnitude of losses (3).

Livestock populations are usually described in terms of the number of animals, yet this metric fails to account for differences in production purpose, breed, and population structure, which contribute to productivity and economic value. Using livestock biomass (i.e., the sum of individual liveweights for a given population) as an alternative allows a comparison of populations with different animal sizes across production systems. Furthermore, quantifying the current productivity (e.g., milk yields) of livestock in different production systems is the first step in understanding yield gaps. Similarly, estimating the economic value of livestock in a production system provides the basis for further work to capture expenditure on animal health. A recent study by Jemberu et al. (4) analyzed the biomass and economic value of small ruminants in production systems in Ethiopia. However, relevant information about cattle production systems in Ethiopia is still lacking.

Cattle make a critical contribution to livelihoods across Ethiopia, and the cattle population has increased from 53 million head in 2010 to 70 million in 2020 in response to increasing demand for livestock products (5). The vast majority (98%) of cattle in Ethiopia are local *Bos indicus* breeds kept by smallholder farmers for draft power and milk production in the mixed crop-livestock (70%) and pastoral (28%) zones (Shapiro 2017). The remaining cattle are crossbred or exotic breeds managed in specialized dairy and beef production systems in urban and peri-urban areas (6).

While the importance of cattle to Ethiopia is well documented, a lack of system-specific information on the biomass, productivity and value of cattle limits our ability to accurately analyze these systems. The aim of this paper is to address these knowledge gaps for the different cattle production systems within Ethiopia. This information will be used as a baseline for further disease burden analysis, which quantifies the scale and cost of production losses.

## 2. Methods

The biomass, productivity and value of cattle for 2021 were calculated for the lowland grazing (pastoral), mixed crop-livestock (CLM) and specialized systems using existing secondary data obtained from national statistics (7–9) and the literature. Analysis of the specialized systems was limited by the available data, and only specialized dairy systems could be considered.

Livestock biomass was calculated as the sum of the liveweight of all cattle within each production system using data on livestock populations, herd dynamics and liveweights. This information was combined with data on productivity and value to calculate livestock outputs and the value of livestock production. Cattle values include the stock value and the output value. In this study, the stock value refers to the total economic value of a cattle population in a system, and it was calculated by the average price of an individual multiply by the total population of the interested population. The output value refers to the total economic values of all the cattle products such as beef, milk and hides. The total value of each output can be calculated by using the total volume multiply by the average price of a product. The biomass and values were calculated by a year basis.

### 2.1. Distribution and characteristics of cattle populations

#### 2.1.1. Total cattle population

Data on the total cattle population in the CLM and pastoral systems was sourced from the 2020/21 agriculture sampling survey of the Central Statistical Agency of Ethiopia (CSA) (9). This survey is the most comprehensive national agricultural survey in Ethiopia and is conducted on an annual basis. The survey reports the total cattle population (by age, sex and breed) and number of cattle holdings in rural areas. The data was aggregated at the zonal level, with zones roughly aligning with the pastoral and CLM production system borders (Supplementary Table S1). Thus, populations and number of farms from each zone were combined to provide the population and number of farms for each production system. This data was then used to calculate the density of cattle in each zone using the ‘tmap’ and ‘sf’ packages in R (version 4.1.3) (10–12), and as the basis for subsequent calculations for biomass and economic value. The CSA data do not report the number of cattle in specialized dairy and beef production systems, and these systems account for approximately 3% of the national cattle population (13). Based on the relative proportion of cattle in feedlots (6, 13), we assumed that 10% of these cattle are in specialized beef systems, with the rest specialized dairy. The distribution of specialized dairy and beef production systems could not be mapped since they are dispersed and not aligned with zones.

#### 2.1.2. Herd size and the number of holdings

For the characteristics of farms, average herd size and the number of holdings of each size within the CLM and pastoral systems were calculated using data from CSA (9). This data was not available for specialized systems. The average number of cattle of different age/sex groups on a farm in the CLM system and the pastoral system were calculated using the LSMS dataset using data from 2018/19 (8). Herd structure was defined as the number of cattle of each sex within specific age groups, where juvenile cattle are calves <1 year old, subadults are cattle aged 1 to 3 years, and adults are cattle >3 years.

#### 2.1.3. Liveweight and parturition rate

Average live body weights and parturition rate (average number of births per reproductive female per year) were estimated for the CLM and pastoral systems using a meta-analysis of published values (Supplementary Section S2). There was insufficient publications on specialized dairy systems to include these in the met-analysis, so information on these systems was taken directly from Shapiro (13). Live body weights of indigenous cattle were used for cattle in the CLM and the pastoral systems. Body weights of a female and a male in a system were assumed the same due to limited sex-specific data in the meta-analysis. We assumed the probability of male and female births in all systems was equal and the net prolificacy rate was 1.

#### 2.1.4. Offtake

Net offtake rates were calculated for each class (age/sex) of cattle in the CLM and the pastoral systems using data from CSA (Equation 1) (9). Offtake rate refers to the proportion of cattle leaving the herd each year due to slaughter, sale, or gifting, and also accounts for animals which enter the herd as gifts or purchases. It does not include the death of animals due to injury or illness, which are calculated separately as the death rate.


(1) $$offtakeof\;a\;\mathrm{group}=\frac{\begin{array}{l} (numberofsale+numberof\;slaughtered\\ +numberofoffered\;as\;gift\\ -numberofpurchased\\ -numberofget\;as\;gift) \end{array}}{totalpopulationofthegroup}$$

For the offtake rates of cattle in the CLM system, the LSMS data was used. For the offtake rates of cattle in the pastoral system, the 2020/21 agriculture sampling survey (8, 9) was used due to data gaps in LSMS data for the pastoral system. Offtake rates of juvenile cattle in the pastoral system was assumed to be zero since slaughter and sale of calves is less popular than that of the other age groups in Ethiopia (14). The offtake rates of cattle in the specialized dairy system were from literature (13).

#### 2.1.5. Mortality

Death rates were also calculated for each class of cattle (Equations 2–5) in the CLM and pastoral systems. Death rates for juvenile cattle in the CLM and the pastoral systems were estimated *via* a meta-analysis (Supplementary Section S2). This analysis did not disaggregate deaths by sex as studies rarely reported sex-specific mortality, and it was assumed that death rates for juvenile cattle were similar in males and females. Then, assuming that subadult and adult cattle contributed equally to the total dead cattle in 2020, the death rates of female subadult and female adult was calculated using data from CSA (9). Cattle mortality in the specialized dairy system was from literature (13).


(2) $$\begin{array}{c} deathrateof\\ femalesubadult=0.25* \end{array}\frac{\begin{array}{l} total\;\mathrm{number of}\;death\\ -total\;\mathrm{number of}\;deathofjuvenile \end{array}}{totalfemalesubadult}$$

(3) $$\begin{array}{c} deathrateof\\ femaleadult=0.25* \end{array}\frac{\begin{array}{l} total\;\mathrm{number of}\;death\\ -total\;\mathrm{number of}\;deathofjuvenile \end{array}}{totalfemaleadult}$$

(4) $$\begin{array}{c} deathrateof\\ malesubadult=0.25* \end{array}\frac{\begin{array}{l} total\;\mathrm{number of}\;death\\ -total\;\mathrm{number of}\;deathofjuvenile \end{array}}{totalmalesubadult}$$

(5) $$\begin{array}{c} deathrateof\\ maleadult=0.25* \end{array}\frac{\begin{array}{l} total\;\mathrm{number of}\;death\\ -total\;\mathrm{number of}\;deathofjuvenile \end{array}}{totalmaleadult}$$

The daily milk outputs of a cow in the CLM and pastoral systems were calculated using data from the national statistics (7). The daily milk output of a cow in zones in a production system was averaged to estimate the daily milk output of a cow in the production system, and the quantiles of 25 and 75% were reported. The daily milk outputs of a cow in the specialized dairy systems were triangulated from literature (6, 15, 16).

### 2.2. Cattle biomass and economic value

Cattle biomass and value in the CLM, pastoral and dairy systems were calculated using Dynmod (17), a spreadsheet-based herd dynamic model used to reflect a population in a steady state with constant growth rate and sex-by-age structure. Dynmod provides annual estimates of livestock biomass and key outputs such as milk, meat, and hides. The model requires input data on population numbers, structure, reproduction rates, mortality, and offtake for the different classes of cattle, as described above. The other parameters such as price of live cattle, skin and hide offtake and manure output were from literature (Supplementary Tables S2–S4). The steady-state model of Dynmod was employed. Population data from 2020 (Table 2) was used as the starting population producing estimates of population and output for 2021. The model was parameterized using point parameter values describing characteristics of livestock populations (Tables 2, 3), and ranges of values and a sensitivity analysis were then applied to illustrate the uncertainties in the biomass estimation and to identify key determinates of accuracy.

The numbers of cattle in age/sex groups in the CLM and the pastoral systems were calculated using the 2020/21 agriculture sampling survey of the CSA (9). The numbers of cattle in age/sex groups in the dairy system were calculated using the proportions of cattle in age/sex groups from literature (21).

To illustrate the uncertainties in the biomass, productivity and economic aspects, values of live body weight, productivity, mortality, and prices from Food and Agriculture Organization (FAO), national statistics and literature were compared (Supplementary Table S5). A Monte Carlo simulation with sensitivity analysis was then performed for cattle biomass estimation of the CLM and the pastoral systems. Variable uncertainty was modeled using probability distributions in ModelRisk (22). For example, a normal distribution was used to show the range of the cattle population size. The mean value was the sum of the mean values in zones of the production system, and the standard error was calculated based on data from the Agricultural Sample survey 2020/21 (7) (Supplementary Sections S5, S6). The 95% confidence intervals (CI) of the cattle biomass in the two production systems were calculated, and the impacts of the uncertainties of variables on the biomass were illustrated.

The economic value was calculated based on the asset and output values calculated by Dynmod. The asset value was taken as the value of live animals and based on the average stock population of cattle in production systems. The prices of cattle of different age/sex groups were from livestock markets surveyed in 2020 (23). Cattle prices are reported as Grade 1 or 2, based on carcass quality. Grade 1 cattle are usually crossbred or imported cattle breeds in good condition, most likely from specialized beef and dairy systems. Most of the cattle from pastoral and CLM systems are sold as Grade 2. Producer prices for beef, milk, draft power, and cattle hides values were not available, so were sourced from literature (Table 1). The total value of the meat, milk and draft power was compared with national GDP in 2021 (110 USD Billion (28)). Output values per kg of biomass were calculated for production systems.

**Table 1:** Value of livestock products.

Product	Price	Source
Beef	4.4 USD/kg	Wamucii (24)
Milk	0.91 USD/L	Gustafson (25)
Draft power	2.6 USD/day for 80 days/year	Shaw et al. (26)
Hides	0.12 USD/kg	Abdu et al. (27)

## 3. Results

### 3.1. Distribution and characteristics of cattle populations

**Figure 1:**
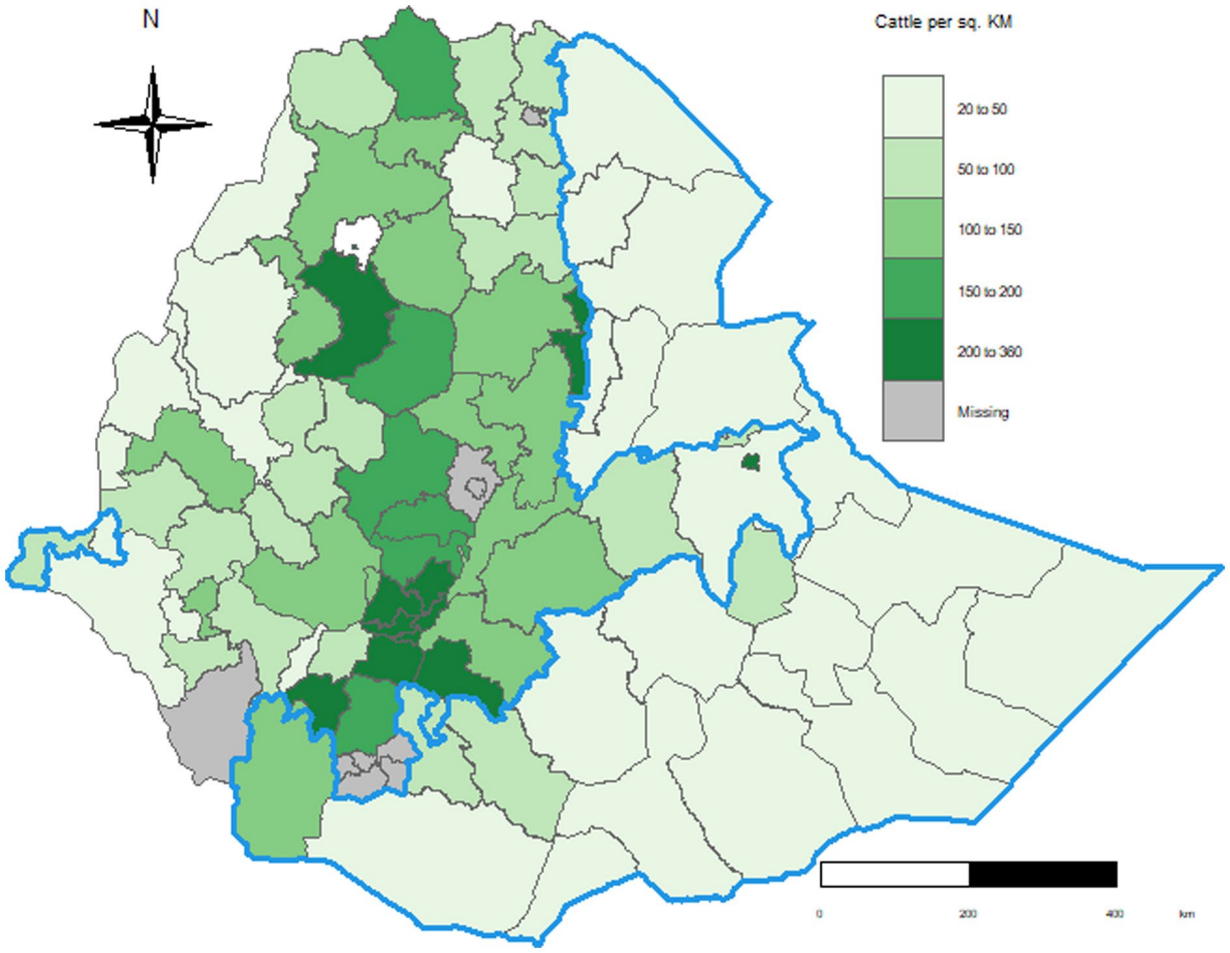
Cattle population density in Ethiopia by zones and production system in 2020/21 (9). The zones within the blue border lines are mostly pastoral cattle production system areas, and the others belong to the mixed crop-livestock system, which are typically highland. The white gap (north-west) is Lake Tana.

In 2021, the cattle population in the Ethiopian mixed crop-livestock, pastoral and specialized dairy systems were 55.0, 13.4 and 1.8 million, respectively. In general, cattle densities in the highland areas of the mixed crop-livestock zone appear to be higher than those in the lowland pastoral regions (Figure 1). The specialized dairy system is not illustrated in the figure below but is primarily located in peri-urban areas in the highland regions.

**Figure 2:**
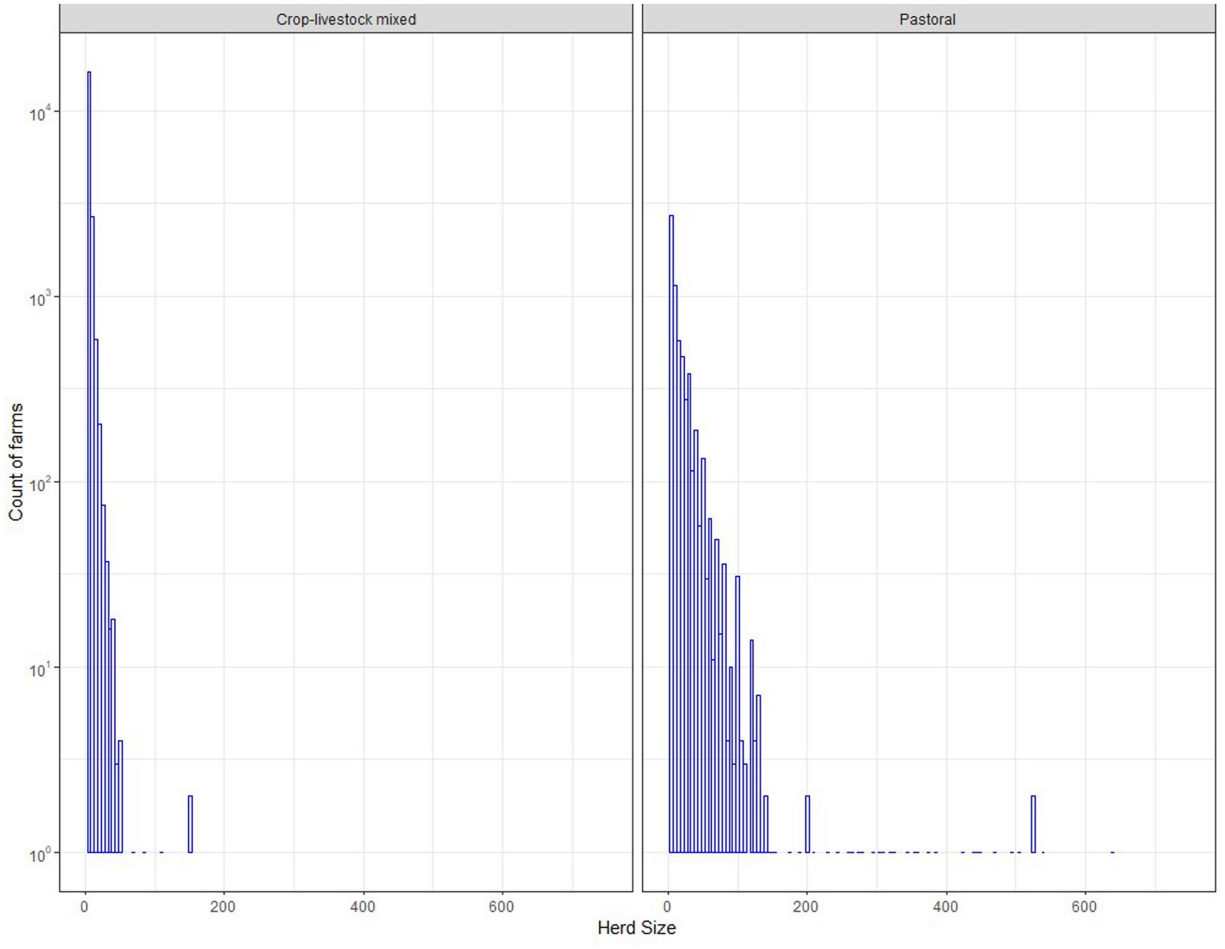
Frequency of herd sizes for households in the crop-livestock production system and the pastoral system in 2020 [data source: Central Statistical Agency of Ethiopia (9)]. This information is not available for specialized dairy production system.

There were 16.8 million and 2.0 million cattle farms in the CLM and the pastoral systems, respectively in 2020/21. Small cattle farms dominate the mixed crop-livestock and pastoral production systems, though larger herds of up to approximately 150 and 750 exist in the mixed crop-livestock and the pastoral production systems, respectively (Figure 2). The average herd size of the farms in the mixed crop-livestock system was 4.4 (median: 3) heads, while the average herd size of the farms in the pastoral system was 15.8 (median: 7) heads. This information is not available for the specialized dairy system.

There were large differences in herd structure between the three systems (Table 2). Cattle farms in the mixed crop-livestock system had the highest proportion of adult males (35%), a large proportion of which are used for draft. In comparison, farms in the specialized dairy system had the lowest proportion of adult males (2%) and the highest proportion of adult females (49%) and sub-adult females (24%) (Supplementary Section S7). Cattle in the specialized dairy systems also had higher average body weights compared to cattle in the mixed crop-livestock and pastoral systems (Table 2).

**Table 2:** Number of cattle and average live body weights of different sex/age cattle groups for different production systems in Ethiopia (9, 13, 18).

Animal class	Population (million head, % of herd)			Average Liveweight (kg, 95% CI of mean)		
	Mixed	Pastoral	Specialized dairy	Mixed	Pastoral	Specialized dairy*
Female Juvenile	4.19 (8%)	2.13 (16%)	0.33 (16%)	83 (63–104)	83 (63–104)	148 (130–165)
Female Subadult	4.47 (8%)	1.36 (10%)	0.44 (21%)	189 (135–242)	189 (135–242)	375 (300–450)
Female Adult	16.7 (30%)	5.32 (40%)	0.91 (44%)	242 (162–323)	242 (162–323)	550 (425–675)
Male Juvenile	4.66 (8%)	1.44 (11%)	0.09 (16%)	83 (63–104)	83 (63–104)	148 (130–165)
Male Subadult	5.48 (10%)	0.76 (6%)	0.05 (2%)	189 (135–242)	189 (135–242)	375 (300–450)
Male Adult	19.5 (35%)	2.44 (18%)	0.04 (2%)	242 (162–323)	242 (162–323)	550 (425–675)

*A range of value was given in the literature for the dairy system, the middle values of the ranges were used as means here.

The average parturition rates in the mixed crop-livestock and the pastoral systems in 2020 were 0.68 (95% CI: 0.48–0.84) and 0.73 (0.59–0.84) per cow per year, respectively. Parturition rates for cows in specialized dairy system are not available for 2020, but are reported by Shapiro (13) as 85–90%. Death rates were higher in the pastoral system compared to the mixed crop-livestock and specialized systems (Table 3). In general, death rates were highest in juvenile animals except for the crop-livestock mixed system (Table 3).

**Table 3:** Death and offtake rates for Ethiopian production systems (9, 13, 19, 20).

	Death rate (95%CI)			Offtake rate		
Animal class	Mixed	Pastoral	Specialized dairy	Mixed	Pastoral	Specialized dairy
Female Juvenile	0.09 (0.04–0.19)	0.29 (0.17–0.44)	0.12 (0.07–0.18)	0.02	0.00	0.11
Female Subadult	0.08	0.16	0.02 (0.00–0.86)	0.02	0.03	0.26
Female Adult	0.02	0.04	0.03 (0.02–0.05)	0.07	0.05	0.28
Male Juvenile	0.09 (0.04–0.19)	0.29 (0.17–0.44)	0.12 (0.07–0.18)	0.02	0.00	0.71
Male Subadult	0.10	0.28	0.02 (0.00–0.86)	0.00	0.02	0.00
Male Adult	0.03	0.09	0.03 (0.02–0.05)	0.09	0.27	0.05

In the crop-livestock mixed and the pastoral systems, offtake of adults was higher than sub adults and juveniles. Tschopp et al. (19) reported that the total offtake rate in the specialized dairy system as 0–71%.

The milk productivity of cows in the mixed crop-livestock (1.45 L/d, Q1-Q3: 1.22–1.68) and pastoral systems (1.79 L/d, Q1-Q3: 1.59–1.72) are broadly similar, with slightly higher daily yields in pastoral cows (9). The average length of lactation is 6.5 months (Q1-Q3: 6–7) and 6.4 months (Q1-Q3: 6–7) in the mixed crop-livestock and pastoral systems, respectively. Cows in the specialized dairy systems have a higher milk productivity of 15–20 L per day and a lactation of 6.7–12.7 months.

### 3.2. Cattle biomass and value

**Figure 3:**
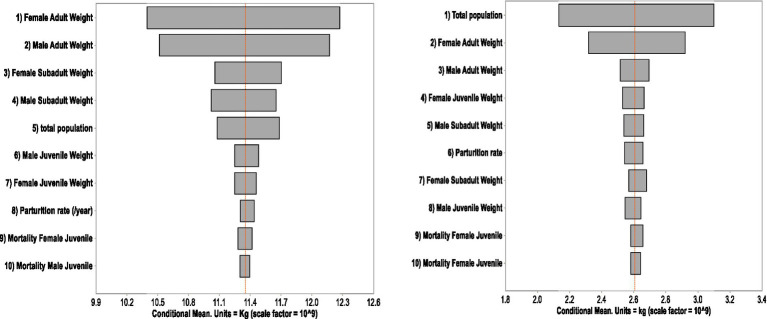
Sensitivity analysis of the variables used for cattle biomass estimation in the crop-livestock mixed (left) and the pastoral (right) systems in Ethiopia.

The total biomass of cattle in the CLM, pastoral, and specialized dairy systems in 2021 were estimated as 11.3, 2.60 and 0.87 billion kg, respectively. Based on the assumed variations of parameters, the total cattle biomass of 2021 in the CLM production system and the pastoral system varied between 9.8–12.9 billion kg (95% CI) and 2.0–3.2 billion kg (95% CI), respectively. The sensitivity analysis showed the rank of the impacts from variation in inputs on the total cattle biomass (Figure 3). For the CLM system, the variability of the average liveweight of an adult female contributed most to the level of uncertainty, followed by liveweight of adult male. Uncertainty in the liveweight of juveniles, parturition rate and calf mortality rates had little impact. For the cattle biomass of the pastoral system, the total population and liveweight of adult cows contributed most to the level of uncertainty (Figure 3).

The total output values of the meat, milk, hides, and draft power from the three production systems were 12.35 billion USD, which contributed 11.2% of GDP in Ethiopia in 2021 (Table 4). The output values per biomass unit (kg) of the CLM, pastoral and specialized dairy systems were 0.75 USD/kg, 0.70 USD/kg and 1.83 USD/kg.

**Table 4:** Productivity, outputs and values of cattle in the crop-livestock mixed system and the pastoral system in Ethiopia in 2021.

Item	Mixed	Pastoral	Specialized
Milk production (billion litre)	2.84	1.41	1.34
Value of milk output (billion USD)	2.59	1.29	1.22
Meat production (million kg)	495	124	84
Value of meat (billion USD)	2.18	0.55	0.37
Skin/hides (million kg)	59.8	2.95	4.06
Value of hides (million USD)	7.36	0.35	0.50
Draft power (billion ox day’s work)	1.45	NA	NA
Value of draft power (billion USD)	3.75	NA	NA
Value of livestock (billion USD)	24.8	5.28	1.37
Manure (billion kg)	23.6	5.55	0.92
Total output value (billion USD)	8.52	1.83	1.59
Output values per kg of biomass (USD/kg)	0.75	0.70	1.83

NA for not applicable.

## 4. Discussion

An understanding of the biomass and values of livestock by production system can benefit the development of good animal health policy. Knowledge of livestock biomass and the economic value of the livestock sector can help policymakers advocate for fair allocation of resources toward improving animal health and productivity. To the best knowledge of the authors, this is the first study describing the cattle biomass and economic value in the three dominant production systems in Ethiopia. Although cattle biomass in Ethiopia was calculated using estimates for cattle population and live weight in a previous study (29), the authors did not consider the variation in live weights of different age/sex groups of cattle, nor did they disaggregate their analysis by production system. The cattle population (25.8 million) has also increased substantially since that study was conducted. The findings from the present study offer insights on the difference of cattle production systems in terms of their population distribution, productivity, and values.

Ethiopian cattle production is constrained by low productivity. The indigenous cattle breed with small live body weights and poor milk productivity are dominant in the cattle population in this country. The vast majority (98%) of cattle in Ethiopia are local *Bos indicus* breeds kept by smallholder farmers for draft power and milk production in the mixed crop-livestock (70%) and pastoral (28%) zones (13). The remaining cattle are crossbred or exotic breeds managed in specialized dairy and beef production systems in urban and peri-urban areas. There is a great potential to improve the productivity in local cattle production systems. For example, the average live weight of a cow in the mixed and pastoral systems is only 242 kg in Ethiopia, while the average live weight of a cow in South Africa can reach to nearly 500 kg (30). Furthermore, in 2021, the annual milk output of an Ethiopian cow was substantially lower than the world average (449.9 kg VS. 2,692 kg) (31). These disparities raise significant concerns regarding the productivity of indigenous cattle breeds and the potential hindrance they pose in meeting the increasing demand for beef and dairy consumption within the country. Addressing the low offtake rates also holds relevance in evaluating the overall efficiency and growth potential of the cattle production systems in this country. It worth mentioning that the low productivity of local cattle was not only due to genetics but also other constrains, such as diseases, poor management and limited feed resources (1, 21, 32).

The output values per kilogram of biomass offer a valuable means of comparing different production systems. Notably, the dairy production system exhibits over twice the output values per kilogram of biomass compared to the other two production systems in Ethiopia, underscoring its higher productivity in this country. Moreover, this metric presents a benchmark for potential enhancements across all production systems, encouraging further efficiency and effectiveness in the livestock sector. Additionally, livestock biomass *per capita* serves as a significant measure of food security status and progress within a country. By assessing the *per capita* biomass, we can gain insights into the availability and utilization of livestock resources, thus influencing strategies for sustainable agricultural development. Furthermore, economic loss per kilogram of biomass emerges as an informative indicator for evaluating the health status of livestock populations. Analyzing economic losses associated with each unit of biomass sheds light on the overall well-being and would allow to compare disease burdens in different production systems and in different countries. The data presented in this study offer a baseline for disease burden analyzes by the GBADs program (2) and further analyzes by other stakeholders. For example, livestock biomass can also be used as an input into analysis of greenhouse gas emissions, land carrying capacity, and antimicrobial usage (33, 34). The GBADs program uses total biomass and economic value of livestock as the first step for disease burden analysis. Biomass is used as the denominator for comparing investments into livestock production, the value of livestock and their products, and the cost of lost production between different species and geographies. Biomass is more useful denominator than livestock population as it accounts for the variation in sizes of different livestock types (e.g., age, breed, species) and therefore also variations in inputs such as feed and antibiotics, and the outputs of products such as meat and milk. For example, Jemberu et al. (4) reported that the total biomass of the small ruminants was 2.1 billion kg in 2020/21 in Ethiopia. This study indicates that cattle biomass was seven times of biomass of small ruminants in the country.

The differences in population composition, reproductivity, productivity and economic values between the pastoral, CLM and specialized systems highlight the need to analyze disease burdens by production system. For example, cattle density was higher in the highland districts of the CLM production system than the lowland districts dominated by the pastoral (Figure 1). This is likely to impact the incidence of diseases spread by direct contact, droplets or airborne transmission (e.g., respiratory infections, foot and mouth disease), with Meadows et al. (35) reporting a positive association between diseases and cattle density. Similarly, differences in herd structure and production purpose will be associated with differences in the types of diseases and scale of economic impact. For example, farms in the CLM production system have the highest proportion of oxen, with draft power provided by these animals contributing almost a third of the total gross output value of livestock in the country (36). Health problems such as lameness that impact the utility of oxen would have a large impact on livelihoods of smallholder farmers who rely on these cattle for draft power. In addition, lameness in oxen in sowing and harvest seasons has potential implications for crop production and income of local farmers. In comparison, the specialized dairy farms have the highest proportion of milking cows, so they are more sensitive to diseases that would impact reproductivity and milk productivity, quality and safety (e.g., brucellosis, mastitis, salmonella).

Data on cattle populations and value reflect the different purposes of production in each system, and when combined with information on small ruminant populations (4) provide insights into the importance of different livestock across Ethiopia. The CLM system has the largest cattle population (i.e., 55 million head, 78% national cattle population in 2021) and cattle biomass (i.e., 11.3 million tonnes, 77% total national biomass in 2021), and the value of cattle production is concentrated in this region (i.e., 8.52 billion USD, 71% of total cattle output value in Ethiopia in 2021) (Table 4). Comparison with the results provided by Jemberu et al. (4) shows that cattle are also the most important livestock species in the CLM, with the annual value of small ruminants in 2021 being roughly one third that of cattle (3.25 billion USD). The dominance of small herd sizes in this system (e.g., cattle 4.4 heads, sheep 3 head, goats 4 head; Figure 2 and Jemberu et al. (4)) indicates the importance of ruminants to smallholder farmers as a source of draft power, income, and food. In the arid lowlands, cattle play a different but again central role in the pastoral system. Larger herd sizes reflect the importance of livestock as bankable assets, and the value of cattle and small ruminants is more evenly distributed (i.e., cattle 1.48 billion USD, sheep 1.02 billion USD, goats 1.69 billion USD) (Table 4 and Jemberu et al. (4)). While the specialized dairy farms only hold about 3% of total cattle population, they contribute 13% of total national value (i.e., 1.59 billion USD, Table 4). This is because of their higher milk yields, driven by the dominance of crossbred and exotic breeds, and better nutritional and health management compared to cattle in the other systems.

Our results also highlight the large variation in reported values for livestock production in Ethiopia. Inconsistent values from different data sources were found when triangulating values for biomass and economic value estimation. For example, different values of the live body weight of Ethiopian cattle can be found in the FAOSTAT, the Domestic Animal Diversity Information System^1^ and literature (13, 31). In this study, we combined meta-analysis and triangulating values from different sources to estimate parameters for biomass and value estimation. The population values were from national statistics which is considered as the most reliable data. However, other parameters such as sex/age specific mortality was not included in the national surveys. It is challenging to determine which values to use when different values from different sources were observed. A systematic assessment of data reliability should be considered when estimating the biomass and economic values. Part of the reason for different values from literature is that the studies were conducted at different levels (e.g., zonal, regional or national) or in different areas or years in the country. Furthermore, different definitions of one variable may exist between various data sources. For example, the age groups of cattle were defined as juvenile, subadult and adult groups in the national Agricultural Sampling Survey, while in the Livestock Market Information System, the age groups were young, immature and mature (9, 23). These definitions need to be carefully checked to see if their definitions match with each other, and good knowledge of local livestock production and official data reporting system is needed for the checking. Clear and transparent definitions of terms from different data recording systems are beneficial. Establishing an ontology of livestock disease burden analysis would reduce obstacles in triangulating variable values for livestock biomass and value estimation.

Uncertainty in biomass estimation was addressed using Monte Carlo simulation in this study. The variation in a variable can be due to the nature of the data generation. Many of the values had been produced by sampling, and they were along with confidence intervals. Sensitivity analysis offers insights into which variables are crucial for the accuracy of biomass estimation. The variables with larger impacts on the variation of the interested values need to be as accurate as possible. Likewise, the estimation of other interested values, such as economic values, would also benefit from a sensitivity analysis. This procedure will save time and resources when quick livestock biomass and value estimation are needed.

Finally, our study also reveals substantial data gaps, especially for the specialized production systems. These gaps include missing data, data which is only available at coarse scales and cannot be disaggregated to production systems, and data that is out of date. Breed structures of production systems were unknown, which reduces accuracy of average liveweight, population biomass and asset value of livestock. Age/sex specific values for farm gate prices were rarely available. The market prices of beef and milk in 2021 in the Ethiopian Livestock Market Information System (23) were used in this study, which may overestimate the output values of cattle. The hire price of an ox in literature was used to calculate the value of draft power, and it may underestimate the real value of it as the price was of 2014. To address these data gaps, assumptions were made in the calculation, which may introduce a bias. For example, equal mortality from male and female calves was assumed when sex-specific mortality was not available.

## 5. Conclusion

The CLM production system contributed much more biomass and output values than the other systems. The herd structures, reproductivity, productivity and value of outputs of the cattle production systems are different. Values from the national statistics, literature and other data sources are able to support the estimation of cattle biomass and value by production systems in this country. However, the variations of the variables would introduce uncertainty in the estimations. The findings of this study show that the mortality, productivity and outputs vary between cattle production systems in Ethiopia. Analyzing animal health burden by production system is therefore necessary to ensure stakeholders have sufficient data for advocacy and decision making around what investments are needed, by whom and where, for most efficient improvements in animal health and production.

## Data availability statement

The original contributions presented in the study are included in the article/Supplementary material, further inquiries can be directed to the corresponding author.

## Author contributions

YL contributed to the conception and design of the study and writing of the first draft of the manuscript. DM, MH, and JR contributed to the conception and design of the study. YL, WJ, and TK-J contributed to acquisition of data, statistical analysis, and interpretation of the data. DM, GC, and PS contributed to revising the manuscript. All authors have read and approved the submitted version of the manuscript.

## Footnotes

^1^DAD-IS, http://dad.fao.org.
