# Supplementary material for: Characterizing Ethiopian cattle production systems for disease burden analysis
Source: Front Vet Sci. 2023 Sep 28;10:1233474. doi: 10.3389/fvets.2023.1233474 (PMC10598381; doi:10.3389/fvets.2023.1233474)
Supplement: Supplementary file 1 [file Data_Sheet_1.doc]

# Supplementary Material

1. List of zones in cattle production systems in Ethiopia
2. Meta-analysis on liveweight, parturition rate and mortality
3. Calculation of cattle biomass and values using Dynmod
4. Values of live body weight, productivity, mortality, and prices were reported in different sources
5. Uncertainty in cattle biomass estimation
6. Monte Carlo simulation on the total biomass and sensitivity analysis using Dynmod
7. Age/sex structure of cattle farms in the crop-livestock mixed system and the pastoral system

# Allocation of zones to production systems

**Supplementary Table 1.** List of zones and regions within Ethiopia and their allocation to mixed crop-livestock and pastoral production systems.

| **Region code** | **Region name** | **Zone name in English** | **Production system** |
| --- | --- | --- | --- |
| 1 | Tigray | North-west | Mixed crop-livestock |
| 1 | Tigray | Central | Mixed crop-livestock |
| 1 | Tigray | East | Mixed crop-livestock |
| 1 | Tigray | South | Mixed crop-livestock |
| 1 | Tigray | West | Mixed crop-livestock |
| 1 | Tigray | South-east | Pastoral |
| 2 | Afar | Zone 1 | Pastoral |
| 2 | Afar | Zone 2 | Pastoral |
| 2 | Afar | Zone 3 | Pastoral |
| 2 | Afar | Zone 4 | Pastoral |
| 2 | Afar | Zone 5 | Pastoral |
| 3 | Amhara | North Gondar | Mixed crop-livestock |
| 3 | Amhara | South Gondar | Mixed crop-livestock |
| 3 | Amhara | North Wollo | Mixed crop-livestock |
| 3 | Amhara | South Wollo | Mixed crop-livestock |
| 3 | Amhara | North Shewa | Mixed crop-livestock |
| 3 | Amhara | East Gojjam | Mixed crop-livestock |
| 3 | Amhara | West Gojjam | Mixed crop-livestock |
| 3 | Amhara | Wag Hemra | Mixed crop-livestock |
| 3 | Amhara | Awi | Mixed crop-livestock |
| 3 | Amhara | Ormo special zone | Mixed crop-livestock |
| 3 | Amhara | Central Gondar | Mixed crop-livestock |
| 3 | Amhara | West Gondar | Mixed crop-livestock |
| 4 | Oromia | West Wollega | Mixed crop-livestock |
| 4 | Oromia | East Wollega | Mixed crop-livestock |
| 4 | Oromia | Illu Ababbor | Mixed crop-livestock |
| 4 | Oromia | Jima | Mixed crop-livestock |
| 4 | Oromia | West Shewa | Mixed crop-livestock |
| 4 | Oromia | North Shewa | Mixed crop-livestock |
| 4 | Oromia | East Shewa | Mixed crop-livestock |
| 4 | Oromia | Arsi | Mixed crop-livestock |
| 4 | Oromia | West Hararghe | Mixed crop-livestock |
| 4 | Oromia | East Hararghe | Mixed crop-livestock |
| 4 | Oromia | Bale | Pastoral |
| 4 | Oromia | Borena | Pastoral |
| 4 | Oromia | South-west Shewa | Mixed crop-livestock |
| 4 | Oromia | Guji | Pastoral |
| 4 | Oromia | West Giji | Mixed crop-livestock |
| 4 | Oromia | Oromial liyu zone | Mixed crop-livestock |
| 4 | Oromia | West Arsi | Mixed crop-livestock |
| 4 | Oromia | Kelem Wollega | Mixed crop-livestock |
| 4 | Oromia | Horo Gudru Wollega | Mixed crop-livestock |
| 4 | Oromia | Buno Bedel | Mixed crop-livestock |
| 5 | Somali | Siti | Pastoral |
| 5 | Somali | Fafan | Pastoral |
| 5 | Somali | Jerer | Pastoral |
| 5 | Somali | Erer | Pastoral |
| 5 | Somali | Korahe | Pastoral |
| 5 | Somali | Shebele | Pastoral |
| 5 | Somali | Dolo | Pastoral |
| 5 | Somali | Afder | Pastoral |
| 5 | Somali | Liben | Pastoral |
| 5 | Somali | Nogob | Pastoral |
| 5 | Somali | Dawa | Pastoral |
| 6 | Benshabgul Gumuz | Metekel | Mixed crop-livestock |
| 6 | Benshabgul Gumuz | Asosa | Mixed crop-livestock |
| 6 | Benshabgul Gumuz | Kemashi | Mixed crop-livestock |
| 6 | Benshabgul Gumuz | Mao Komo | Mixed crop-livestock |
| 7 | SNNP | Guragie | Mixed crop-livestock |
| 7 | SNNP | Hadiya | Mixed crop-livestock |
| 7 | SNNP | Kembata Tembaro | Mixed crop-livestock |
| 7 | SNNP | Sidam | Mixed crop-livestock |
| 7 | SNNP | Gedeo | Mixed crop-livestock |
| 7 | SNNP | Wollayta | Mixed crop-livestock |
| 7 | SNNP | South Omo | Pastoral |
| 7 | SNNP | Sheka | Mixed crop-livestock |
| 7 | SNNP | Kefa | Mixed crop-livestock |
| 7 | SNNP | Gamo Gofa | Mixed crop-livestock |
| 7 | SNNP | Benchi Maji | Mixed crop-livestock |
| 7 | SNNP | Yem special distr | Mixed crop-livestock |
| 7 | SNNP | Segen Akababi peoples | Mixed crop-livestock |
| 7 | SNNP | Alaba special distr | Mixed crop-livestock |
| 7 | SNNP | Dawuro | Mixed crop-livestock |
| 7 | SNNP | Basketo special distric | Mixed crop-livestock |
| 7 | SNNP | Konta special district | Mixed crop-livestock |
| 7 | SNNP | Siltie | Mixed crop-livestock |
| 12 | Gambella | Agnuwak | Mixed crop-livestock |
| 12 | Gambella | Nuware | Pastoral |
| 12 | Gambella | mezhengir | Mixed crop-livestock |
| 12 | Gambella | Itang special district | Pastoral |
| 13 | Harrerie | Harrerie | Mixed crop-livestock |
| 14 | Addis Ababa | Addis Ababa | Mixed crop-livestock |

# 2. Meta-analysis of liveweight, parturition rate and mortality

These parameters were chosen to conduct meta-analysis due to that they are the key parameters in the Dynmod model, and there are enough literature for a meta-analysis. Pubmed and Scopus were used to look for potential useful literature. Keywords were established to address these aspects: Species, country and topic of interest. Only publications of the past 10 years were searched to make sure the data was not out of date. Details in methods are as follow.

Meta analysis on cattle Mortality in Ethiopia.

Search strategy

PubMed

  (cattle [Title/Abstract]) AND (Ethiopia*[Title/Abstract]) AND ((mortality[Title/Abstract]) OR (death rate[Title/Abstract]) OR (loss [Title/Abstract]) OR (offtake [Title/Abstract])) NOT (virus[Title]) NOT (infect*[Title]) NOT (risk factor*[Title]) AND (y_10[Filter])

Scopus

  TITLE-ABS-KEY ( ethiopia* ) AND TITLE-ABS-KEY ( cattle ) AND TITLE-ABS-KEY ( mortality OR "death rate" OR loss OR death ) ) AND PUBYEAR > 2012 AND PUBYEAR > 2012

Search results

- pubmed N=48
- SCOPUS N=172;
- Deduplicated pubmed and SCOPUS searches N=35;

Two papers were added via extend reading.

Total 185 papers.

Data extraction items

Data Extraction were conducted in 2 sifts. In the first sift, reviewers decide if to include a paper for the next sift according to the following information:

- What was the topic of the study? Only study focus on cattle mortality will be included.
- Does the study a review or a survey? Review excluded.
- Dose it specify the production systems of the mortality? Exclude if not.
- What is the design of these? Outbreak case report, case-control study, disease prevalence and risk factors were not included. A survey needs to be with proper sampling design? Is the Mortality under disease condition should not be considered

21 papers were included in sift two and they were reviewed by reading full text. The following information were extracted from 13 papers:

- What were the production system?
- What were the age group
- What were the species: indigenous, cross-breed or exotic?
- Mortality over how long?
- Year of study: the middle year was taken for a study that covered multiple years

 Assumptions were made when extracting values:

- mortality of 6 months were doubled to make 1-year mortality;
- cumulative mortality observed in 3 years: calve mortality was taken as 1-year mortality when the authors defined cattle age <1-year as calves. However, for subadult and adult, values were divided by 3 to get annual values.

Meta analysis on cattle parturition rate in Ethiopia.

Search strategy

PubMed

Search: (cattle[Title] OR bovine[Title] OR calve[Title] OR calf[Title] OR heifer[Title]) AND (Ethiopia*[Title/Abstract]) AND (patur*[Title/Abstract] OR "reproduct* rate"[Title/Abstract] OR birth [Title/Abstract] OR gestation [Title/Abstract] OR "calving rate" [Title/Abstract] OR "fertility rate" [Title/Abstract]) AND (y_10[Filter])

Scopus

  ( TITLE-ABS-KEY ( ethiopia* ) AND TITLE-ABS-KEY ( cattle ) AND TITLE-ABS-KEY ( "patur*" OR "reproduct*" OR "birth" OR "gestation" OR "calving rate" OR "fertility rate" ) ) AND PUBYEAR > 2012

Search results

- PubMed N=15
- SCOPUS N=72;
- Deduplicated PubMed and SCOPUS searches N=14;

Total 73 papers were identified.

Data extraction items

Data Extraction were conducted in 2 sifts. In the sift one, reviewers decide if to include a paper for the next sift according to the following information:

- What was the topic of the study? Only study focus on cattle parturition rate will be included.
- Excluded studies that only report diseases and their impacts on parturition rate.
- Dose it specify the production systems? Exclude if not.
- What is the design of these? Case report and studies with on-representative sampling were not considered.

18 papers were included in sift two and they were reviewed by reading full text. The following information were extracted from 16 papers:

- What were the production systems?
- What were the breeds: indigenous cross-breed or exotic?
- Farm-type: small holder? Commercial?
- Parturition rate in a year? Mean and SEs of this value.
- Year of study: the middle year was taken for a study that covered multiple years

 Assumptions were made: assuming 100% prolificacy rate as numbers and calculate the annual parturition rate = calve interval in months /12 months.

Meta analysis on cattle live weight in Ethiopia.

Search strategy

PubMed

("cattle"[Title] OR "bovine"[Title] OR "beef"[Title] OR "dairy"[Title] OR "ox"[Title] OR "steer"[Title] OR "calve"[Title] OR "calf"[Title] OR "heifer"[Title]) AND "ethiopia*"[Title/Abstract] AND ("body weight"[Title/Abstract] OR "live weight"[Title/Abstract] OR "mass"[Title/Abstract] OR "weight gain"[All Fields] OR "body conditions"[Title/Abstract]) AND "2013/02/19 00:00":"3000/01/01 05:00"[Date - Publication]

Scopus

  ( TITLE-ABS-KEY ( ethiopia* ) AND TITLE-ABS-KEY ( cattle ) AND TITLE-ABS-KEY ( "patur*" OR "reproduct*" OR "birth" OR "gestation" OR "calving rate" OR "fertility rate" ) ) AND PUBYEAR > 2012

Search results

- PubMed N=14
- SCOPUS N=36
- Deduplicated PubMed and SCOPUS searches N=13

Two papers were added via extended reading.

Total 39 papers for screening

Exclusion on papers if:

- The study doesn’t analyse cattle weight
- Not specify the age/sex groups
- Diseases and their impacts only
- Not representative sampling
- No statistical analysis was conducted
- Clinical trait for exotic/crossbreed only because they are not under farm condition

Data extraction items

32 papers were included in sift two and they were reviewed by reading full text. The following information were extracted from 10 papers:

- Average live body weights: Mean and SEs of this value.
- What were the production system?
- What were the species: indigenous, crossbreed or exotic?
- Age/sex groups studied?
- Year of study: the middle year was taken for a study that covered multiple years

 Assumptions were made: A male and a female cattle in the same age groups of a system had a same body weight where no sex-specific values were reported.

1. Code for Meta-analysis in R: https://github.com/vetlee2/GBADsLiverpool_ly

# 3. Calculation of cattle biomass and values using Dynmod

The reproductivity, mortality, offtake and live body weight of age groups were triangulated from different data sources, including national surveys, literature, project reports and expert opinions (Supplementary Table 2-4). The value of the draught power in the mixed crop-livestock system was calculated using the outputs from the Dynmod model. The proportion of oxen in the adult cattle group was estimated as 48% using the data from the Agricultural sample survey 2020. The working days per oxen per year was 80, and the price of one oxen-day was 2.6 USD.

**Supplementary Table 2.** Parameters of the crop-livestock mixed system

| **Section** | **Parameter** | **Fixed value** | **Data sources** |
| --- | --- | --- | --- |
| Meat | Carcass yield | 0.5 | (Shapiro, 2017) |
| Offtake | Female Juvenile (USD/head) | 210 | Dataset of market prices of livestock, shared by MoA, Ethiopia |
|  | Female Subadult (USD/head) | 320 | Dataset of market prices of livestock, shared by MoA, Ethiopia |
|  | Female Adult (USD/head) | 442 | Dataset of market prices of livestock, shared by MoA, Ethiopia |
|  | Male Juvenile (USD/head) | 181 | Dataset of market prices of livestock, shared by MoA, Ethiopia |
|  | Male Subadult (USD/head) | 390 | Dataset of market prices of livestock, shared by MoA, Ethiopia |
|  | Male Adult (USD/head) | 724 | Dataset of market prices of livestock, shared by MoA, Ethiopia |
| Milk | Lactation length (day) | 210 | Agricultural sample survey 2020 |
|  | Yield (litre per day) | 1.45 | Agricultural sample survey 2020 |
| Skin and hides (kg per offtake) | Female Juvenile | 0 |  |
|  | Female Subadult | 10 | Proportional to the ratio of live body weights of sub-adult and adult |
|  | Female Adult | 15 | fresh weight from FAOSTAT 2019 Ethiopia |
|  | Male Juvenile | 0 |  |
|  | Male Subadult | 10 | Proportional to the ratio of live body weights of sub-adult and adult |
|  | Male Adult | 15 | fresh weight from FAOSTAT 2019 Ethiopia |
| Manure per day (kg) | Juvenile | 0.5 | default value; Proportional to the ratio of live body weights of Juvenile and adult |
|  | Subadult | 0.8 | default value; Proportional to the ratio of live body weights of sub-adult and adult |
|  | Adult | 1.5 | default value; LR (Behnke, 2010) |

**Supplementary Table 3.** Parameters of the pastoral system

| **Section** | **Parameter** | **Fixed value** | **Data sources** |
| --- | --- | --- | --- |
| Meat | Carcass yield | 0.5 | (Shapiro, 2017) |
| Offtake | Female Juvenile (USD/head) | 210 | Dataset of market prices of livestock, shared by MoA, Ethiopia |
|  | Female Subadult (USD/head) | 320 | Dataset of market prices of livestock, shared by MoA, Ethiopia |
|  | Female Adult (USD/head) | 442 | Dataset of market prices of livestock, shared by MoA, Ethiopia |
|  | Male Juvenile (USD/head) | 181 | Dataset of market prices of livestock, shared by MoA, Ethiopia |
|  | Male Subadult (USD/head) | 390 | Dataset of market prices of livestock, shared by MoA, Ethiopia |
|  | Male Adult (USD/head) | 724 | Dataset of market prices of livestock, shared by MoA, Ethiopia |
| Milk | Lactation length (number of days) | 210 | Agricultural sample survey 2020 |
|  | yield (l per day) | 1.79 | Agricultural sample survey 2021 |
| Skin and hides (kg per offtake) | Female Juvenile | 0 |  |
|  | Female Subadult | 10 | Proportional to the ratio of live body weights of sub-adult and adult |
|  | Female Adult | 15 | fresh weight from FAOSTAT 2019 Ethiopia |
|  | Male Juvenile | 0 |  |
|  | Male Subadult | 10 | Proportional to the ratio of live body weights of sub-adult and adult |
|  | Male Adult | 15 | fresh weight from FAOSTAT 2019 Ethiopia |
| Manure per day (kg) | Juvenile | 0.5 | default value; Proportional to the ratio of live body weights of juvenile and adult |
|  | Subadult | 0.8 | default value; Proportional to the ratio of live body weights of sub-adult and adult |
|  | Adult | 1.5 | default value; LR (Behnke, 2010) |

**Supplementary Table 4.** Parameters of the specialized dairy system

| **Section** | **Parameter** | **Fixed value** | **Data sources** |
| --- | --- | --- | --- |
| Meat | Carcass yield | 0.5 | (Shapiro, 2017) |
| Offtake | Female Juvenile (USD/head) | 220 | Dataset of market prices of livestock, shared by MoA, Ethiopia |
|  | Female Subadult (USD/head) | 338 | Dataset of market prices of livestock, shared by MoA, Ethiopia |
|  | Female Adult (USD/head) | 527 | Dataset of market prices of livestock, shared by MoA, Ethiopia |
|  | Male Juvenile (USD/head) | 338 | Dataset of market prices of livestock, shared by MoA, Ethiopia |
|  | Male Subadult (USD/head) | 365 | Dataset of market prices of livestock, shared by MoA, Ethiopia |
|  | Male Adult (USD/head) | 930 | Dataset of market prices of livestock, shared by MoA, Ethiopia |
| Milk | Lactation length (day) | 255 | Minten et al. (2020) |
|  | Offtake (l per day) | 18 | Food and Agriculture Organization of the United Nations (2018) |
| Skin and hides (kg per offtake) | Female Juvenile | 0 |  |
|  | Female Subadult | 10 | Proportional to the ratio of live body weights of sub-adult and adult |
|  | Female Adult | 15 | fresh weight from FAOSTAT 2019 Ethiopia |
|  | Male Juvenile | 0 |  |
|  | Male Subadult | 10 | Proportional to the ratio of live body weights of sub-adult and adult |
|  | Male Adult | 15 | fresh weight from FAOSTAT 2019 Ethiopia |
| Manure per day (kg) | Juvenile | 0.5 | default value; Proportional to the ratio of live body weights of juvenile and adult |
|  | Subadult | 0.8 | default value; Proportional to the ratio of live body weights of sub-adult and adult |
|  | Adult | 1.5 | default value; LR (Behnke, 2010) |

# 4. Variations of biomass, productivity, and value parameters from different sources

To illustrate the uncertainties in the biomass, productivity and economic aspects, values of live body weight, productivity, mortality, and prices from FAO, national statistics and literature were compared. For the FAO data, the average live weight, milk productivity, prices and offtake were taken from FAOSTAT or calculated using FAOSTAT datasets and the Domestic Animal Diversity Information System (DAD-IS) datasets (Food and Agriculture Organization of the United Nations, 2022). For the national livestock statistics, the data were from the 2020 national survey and the LSMS dataset (Central Statistical Agency of Ethiopia, 2021a, b). When the values were presented in the data sources, the used values were listed in table x. When the values, such as parturition, offtake, and death rates, were not presented directly, the data of cows, slaughtered animals and dead animals were used to calculate these values. The granularity from different sources were also compared.

The result shows that different values of live body weight, productivity, mortality, and prices were reported in different sources (Supplementary Table 5). Between different data sources, variations of parturition rate and milk productivity are small, while larger difference were seen for offtake and prices. In addition, the data granularity from different sources were different. For example, data from FAO was often aggregated at the national level with no production system-specific values available. Besides, parameters were sometimes presented differently. For example, milk production data were presented in different time units.

**Supplementary Table 5.** Data on live weight, productivity, mortality, and prices from different sources

| **Item** | **FAO data of 2020** | **National statistics** | **Other Literature** |
| --- | --- | --- | --- |
| Live body weight | 250-271 kg  No age/sex specific values provided  (Food and Agriculture Organization of the United Nations. Statistics Division., 2021)  (Food and Agriculture Organization of the United Nations, 2022) | No data | Production system-specific and age/sex-specific values are between 83-550 kg  (Bayou et al., 2015; Tesfa et al., 2016; Bekele et al., 2017; Shapiro, 2017; Gathura et al., 2020; Abera et al., 2021; Shumye, 2021; Goshu, 2022; Kassahun et al., 2022). |
| Milk productivity (per head) | 1.70 L per day in a year  No production system-specific values provided  (FAO, 1997; Food and Agriculture Organization of the United Nations. Statistics Division., 2021); | Mixed crop-livestock system: 1.45 L/d and lactation of 5-7 months;  Pastoral system: 1.79 L/day and lactation of 5-7 months  (Central Statistical Agency of Ethiopia, 2021b) | Production system-specific values are between 240-380 L per lactation in the CLM and pastoral systems; 10-20 L per day for a cow in the specialised dairy farms  (Lobago et al., 2007; Ayenew et al., 2009; Shapiro, 2017). |
| Parturition rate | No data | Mixed crop-livestock system: 56%; Pastoral system: 69%  (Central Statistical Agency of Ethiopia, 2021b) | Mixed crop-livestock system: 57%; Pastoral system: 65%  (Shapiro, 2017). |
| Mortality | No data | Production system-specific and age/sex-specific values are between 1-28%  (Central Statistical Agency of Ethiopia, 2021b) | Production system-specific and age/sex-specific values are between 5-12%  (Fentie, 2016; Shapiro, 2017; Tschopp et al., 2021). |
| Offtake | 5.6%  No production system-specific and age/sex-specific values provided  (FAO, 1997; Food and Agriculture Organization of the United Nations. Statistics Division., 2021). | Production system-specific and age/sex-specific values are between -2-19%  (Central Statistical Agency of Ethiopia, 2021b) | Production system-specific and age/sex-specific values are between 0-71%  (Shapiro, 2017; Tschopp et al., 2021). |
| Prices of livestock (USD per head) | 205  No production system-specific and age/sex-specific values provided  (FAO, 1997; Food and Agriculture Organization of the United Nations. Statistics Division., 2021). | Age/sex-specific values are between 67-268  (Central Statistical Agency of Ethiopia, 2021b) | Age/sex-specific values are between 181-724  (Shapiro, 2017). |
| Prices of milk (USD per litre) | 0.57*  (FAO, 1997) | No data | 0.91  (Numbeo, 2021) |

* data of 2018 due to that data of 2020 was not available.

# 5. Uncertainty in cattle biomass estimation

Parameters used in Dynmod were estimated using different approaches. Uncertainty around input and output values can be incorportated. To do this Dynmod input parameter values were defined as distributions. ModelRisk software was then used to calculate uncertainty distributions for the output estimates of biomass. This was done using Monte Carlo simulation with 5000 simulations. Sensitivity analysis was then performed to describe how variation in input parameters influenced the biomass estimate (Vose D, 2021).

**Subheading within Dynmod methods – Defining input parameter distributions**

## Uncertainty of cattle population

The cattle populations by zone were from a CSA report. See Annex Table 1 in the Agricultural Sample survey 2020/21 (Central Statistical Agency of Ethiopia, 2021a).

The SE of the total population in each production system was calculated as follows:

Where,

Y: estimated total of specific livestock in a system

$\hat{Y}_{h}$: estimated total of specific livestock in stratum (zone) h

h: the stratum (zones) in the production system

L the number of zones in a system

For the standard error of the estimate of the total livestock population in a production system:

$$SE= \sqrt{Var(Y)}$$

Thus, the total cattle populations and their SE are: 55,047,667 heads of cattle (total) and 812,344 (SE) for the CLM system and 13,443,444 heads of cattle (total) and 1,363,824(SE) for the pastoral system. The cattle population in a production system was defined as a normal distribution with mean and SD (equal to SE).

## Uncertainty of cattle reproduction rate in different production systems

The uncertainty of the parturition rate of a system was estimated from the literature (Shapiro, 2017). The values were modelled using a uniform distribution, as shown in Supplementary Table 6.

**Supplementary Table 6.** Reproduction performance parameters

| Parameters | CLM | Pastoral | Source/ remark |
| --- | --- | --- | --- |
| Parturition rate (%) | Triangle (0.48,0.68, 0.58) | Triangle (0.59,0.73, 0.84) | Literature (Shapiro, 2017) |

Note: Triangle means triangular distribution used here, and the values in brackets refer to the minimum, mode and maximum values

## Uncertainty of cattle mortality in age groups in different production systems

The calf mortality was from a study on mortalities of young animals (Fentie, 2016). The mortalities of calves in CLM and pastoral systems were reported as 9.2-14% and 26-29.2%, respectively. Assuming the same mortality in male and female calves, the calf mortalities in the two systems were modelled using a Pert distribution shown in Supplementary Table 7. To simplify the model, fixed values were used for the mortality values in other sex age groups.

**Supplementary Table 7.** Annual mortality of calves

| Parameters | CLM | Pastoral | Source/ remark |
| --- | --- | --- | --- |
| Female | Triangle  (0.04,0.09, 0.19) | Triangle (0.17,0.29, 0.44) | Literature (Fentie, 2016) |
| Male | Triangle  (0.04,0.09, 0.19) | Triangle (0.17,0.29, 0.44) | Literature (Fentie, 2016) |

Note: Triangle means triangular distribution used here, and the values in brackets refer to the minimum, mode and maximum values

## Uncertainty of live body weight in different production systems

For demonstrating purpose, the body weights of Ethiopian cattle used here were from (Shapiro, 2017). These authors provide ranges of live weights for different sex/age groups. In cases where only one value was given for a sex/age group, a 2kg variation to the value was assumed. The live weights of cattle in different age groups in a production system were modelled using a uniform distribution shown in Supplementary Table 8.

**Supplementary Table 8.** Range of modelled live body weights (kg) of cattle in age/sex groups.

|  | **CLM** | **Pastoral** | **Source/ remark** |
| --- | --- | --- | --- |
| Female Juvenile | Triangle  (63, 83, 104) | Triangle  (63, 83, 104) | Meta-analysis (Bayou et al., 2015; Tesfa et al., 2016; Bekele et al., 2017; Shapiro, 2017; Gathura et al., 2020; Abera et al., 2021; Shumye, 2021; Goshu, 2022; Kassahun et al., 2022). |
| Female Subadult | Triangle  (135, 189, 242) | Triangle  (135, 189, 242) |  |
| Female Adult | Triangle  (162, 242, 323) | Triangle  (162, 242, 323) |  |
| Male Juvenile | Triangle  (63, 83, 104) | Triangle  (63, 83, 104) |  |
| Male Subadult | Triangle  (135, 189, 242) | Triangle  (135, 189, 242) |  |
| Male Adult | Triangle  (162,242, 323) | Triangle  (162, 242, 323) |  |

Note: Triangle means triangular distribution used here, and the values in brackets refer to the minimum, mode and maximum values

# 6. Monte Carlo simulation on the total biomass and sensitivity analysis using Dynmod

The total cattle biomass in the CLM system was used to illustrate the analytical procedure. The inputs in the spreadsheet were defined as previously described using the tool "Select Distribution", and then they were marked as inputs using the function "Output/Input" of the ModelRisk. The "Avgliv. stock" (average livestock population) in the "Live weight equivalent" section of the results was marked as output. With 1000 iterations, the 95% conference interval of the total cattle biomass was reported.

The sensitivity analysis was conducted to show the impacts of the uncertainties of variables to the final biomass estimation. The rank of the impacts of the inputs' variations on the total cattle biomass was illustrated by a tornado plot.

# 7. Age and sex structure of cattle farms in the mixed crop-livestock and pastoral systems


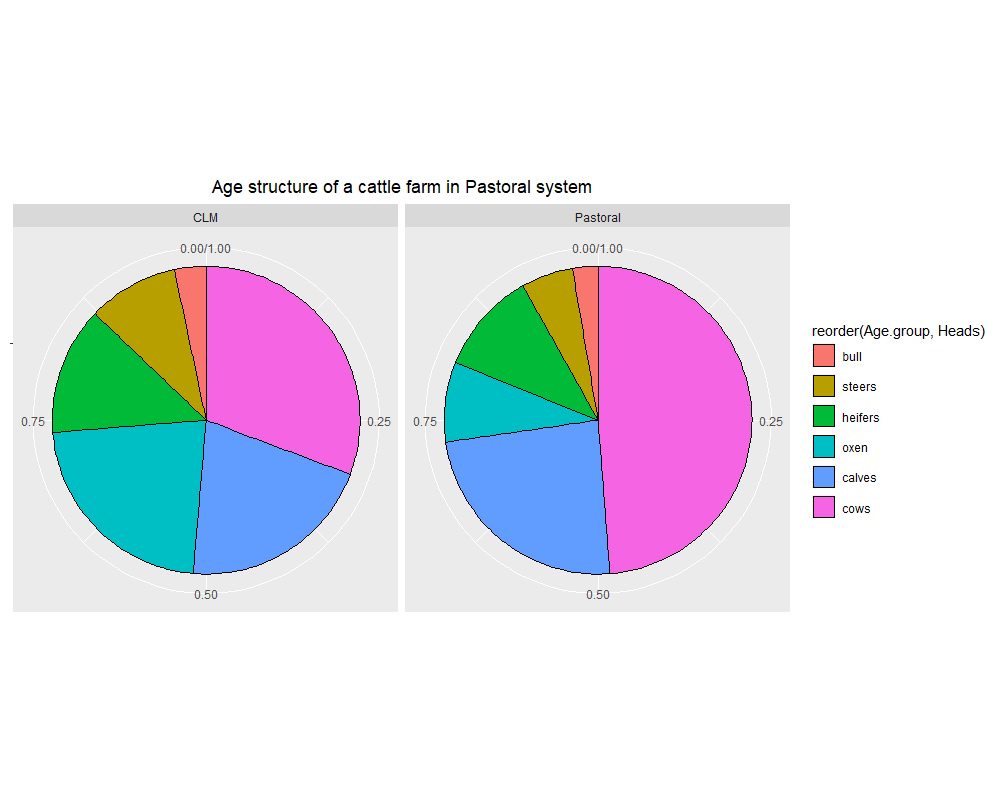


**Supplementary Figure 1**. Age/sex structure of cattle farms in the crop-livestock mixed system (left) and the pastoral system (right)

# References cited in the supplementary section

Abera, M., Eshetu, M., Mummed, Y.Y., Pilla, F., Wondifraw, Z., 2021. Impact of climatic variability on growth performance of Fogera cattle in Northwestern Ethiopia. Journal of Animal Behaviour and Biometeorology 9.

Ayenew, Y.A., Wurzinger, M., Tegegne, A., Zollitsch, W., 2009. Performance and limitation of two dairy production systems in the North western Ethiopian highlands. Trop Anim Health Prod 41, 1143-1150.

Bayou, E., Haile, A., Gizaw, S., Mekasha, Y., 2015. Evaluation of non-genetic factors affecting calf growth, reproductive performance and milk yield of traditionally managed Sheko cattle in southwest Ethiopia. Springerplus 4, 568.

Behnke, R.H., 2010. The contribution of livestock to the economies of IGAD member states: Study findings, application of the methodology in Ethiopia and recommendations for further work. In.

Bekele, A., Wuletaw, Z., Haile, A., Gizaw, S., Mekuriaw, G., 2017. Genetic parameters for reproduction traits and correlation with pre weaning growth traits of Fogera cattle at Metekel ranch, north west Ethiopia. Livestock Research for Rural Development 29.

Central Statistical Agency of Ethiopia, 2021a. AGRICULTURAL SAMPLE SURVEY 2020/21. Central Statistical Agency of Ethiopia, Addis Ababa.

Central Statistical Agency of Ethiopia, 2021b. Ethiopia Socioeconomic Survey (ESS4) 2018-2019.

FAO, 1997. FAOSTAT statistical database. FAO, Rome.

Fentie, T.T., W., Melaku, A., Assefa, G., Tesfaye, S., Fufa, F., et al., , 2016. Assessment of young stock mortality in major livestock production systems of Ethiopia.

Food and Agriculture Organization of the United Nations, 2018. Africa Sustainable Livestock (ASL) 2050: Livestock production systems spotlight – Ethiopia. Rome, Italy, 12.

Food and Agriculture Organization of the United Nations, 2022. Domestic Animal Diversity Information System. FAO.

Food and Agriculture Organization of the United Nations. Statistics Division., 2021. Technical conversion factors for agricultural commodities. Food and Agriculture Organization of the United Nations Rome,.

Gathura, D.M., Muasya, T.K., Kahi, A.K., 2020. Meta-analysis of genetic parameters for traits of economic importance for beef cattle in the tropics. Livest Sci 242.

Goshu, H.A., 2022. Estimation of nonadditive and additive genetic effects on growth traits of Horro cattle. Tropical Animal Health and Production 54.

Kassahun, D., Taye, M., Kebede, D., Tilahun, M., Tesfa, A., Bitew, A., Kebede, A., Meseret, M., Lakew, E., Bimrow, T., Haile, A., 2022. Phenotypic and genetic parameter estimates for early growth, growth rate and growth efficiency-related traits of Fogera cattle in Ethiopia. Vet Med Sci 8, 387-397.

Lobago, F., Bekana, M., Gustafsson, H., Kindahl, H., 2007. Longitudinal observation on reproductive and lactation performances of smallholder crossbred dairy cattle in Fitche, Oromia region, central Ethiopia. Trop Anim Health Prod 39, 395-403.

Minten, B., Habte, Y., Tamru, S., Tesfaye, A., 2020. The transforming dairy sector in Ethiopia. PLoS One 15, e0237456.

Numbeo, 2021. Cost of Living in Ethiopia. Numbeo, Serbia.

Shapiro, B.I., Gebru, G., Desta, S., Negassa, A., Nigussie, K., Aboset G., Mechale. H. , 2017. Ethiopia livestock sector analysis. ILRI Project Report. International Livestock Research Institute (ILRI). Nairobi, Kenya.

Shumye, M., 2021. Growth curve analysis of body weight in crossbred dairy cattle in central Ethiopia. Livestock Research for Rural Development 33, 1-10.

Tesfa, A., Kumar, D., Abegaz, S., Mekuriaw, G., Bimerew, T., Kebede, A., Bitew, A., Ferede, Y., Mazengia, H., Tilahun, M., 2016. Growth and reproductive performance of fogera cattle breed at andassa livestock research center. Livestock Research for Rural Development 28.

Tschopp, R., Gemechu, G., Wood, J.L.N., 2021. A Longitudinal Study of Cattle Productivity in Intensive Dairy Farms in Central Ethiopia. Front Vet Sci 8, 698760.

Vose D, 2021. Risk analysis. Vose Software, Belgium.
